# Ecdysone signaling induces two phases of cell cycle exit in *Drosophila* cells

**DOI:** 10.1242/bio.017525

**Published:** 2016-10-13

**Authors:** Yongfeng Guo, Kerry Flegel, Jayashree Kumar, Daniel J. McKay, Laura A. Buttitta

**Affiliations:** 1 Department of Molecular, Cellular and Developmental Biology, University of Michigan, Ann Arbor, MI 48109, USA; 2 Biology Department and Genetics Department, Integrative Program for Biological and Genome Sciences, University of North Carolina, Chapel Hill, Chapel Hill, NC 27599, USA

**Keywords:** Drosophila, Cell cycle, Ecdysone, Metamorphosis, Steroid hormone

## Abstract

During development, cell proliferation and differentiation must be tightly coordinated to ensure proper tissue morphogenesis. Because steroid hormones are central regulators of developmental timing, understanding the links between steroid hormone signaling and cell proliferation is crucial to understanding the molecular basis of morphogenesis. Here we examined the mechanism by which the steroid hormone ecdysone regulates the cell cycle in *Drosophila*. We find that a cell cycle arrest induced by ecdysone in *Drosophila* cell culture is analogous to a G2 cell cycle arrest observed in the early pupa wing. We show that in the wing, ecdysone signaling at the larva-to-puparium transition induces Broad which in turn represses the cdc25c phosphatase String. The repression of String generates a temporary G2 arrest that synchronizes the cell cycle in the wing epithelium during early pupa wing elongation and flattening. As ecdysone levels decline after the larva-to-puparium pulse during early metamorphosis, Broad expression plummets, allowing String to become re-activated, which promotes rapid G2/M progression and a subsequent synchronized final cell cycle in the wing. In this manner, pulses of ecdysone can both synchronize the final cell cycle and promote the coordinated acquisition of terminal differentiation characteristics in the wing.

## Abstract (precis)

**Summary:** Pulsed ecdysone signaling remodels cell cycle dynamics, causing distinct primary and secondary cell cycle arrests in *Drosophila* cells, analogous to those observed in the wing during metamorphosis.

## INTRODUCTION

Steroid hormones play a central role in coordinating the timing of developmental events. The insect steroid hormone ecdysone is a critical regulator of developmental transitions in hemi- and holometabolous insects, and has long served as a model to study the mechanisms by which steroid hormones control developmental timing (Yamanaka et al., 2013). The ecdysone nuclear hormone receptor signaling pathway is most closely related to the retinoic acid signaling pathway in vertebrates, which also acts a key modulator of cell differentiation in many cell types (Breitman et al., 1980). A pulse of ecdysone signaling occurs during the initiation of metamorphosis at the larva-to-puparium transition (Ashburner, 1989), where extensive changes in proliferation, cell shape, apoptosis and cell adhesion take place. For example, the strong ecdysone pulse at the larva-to-puparium transition leads to activation of apoptotic programs in many larval cell types such as the salivary gland, muscle and midgut (Jiang et al., 1997, 2000; Lee et al., 2002; Yin and Thummel, 2004; Zirin et al., 2013) while in other tissues such as the abdominal histoblasts or dorsal adult progenitor (DAP) cells of trachea, proliferation of adult progenitors is triggered (Djabrayan et al., 2014; Ninov et al., 2009). In the case of abdominal histoblasts, these cells remain quiescent in the G2 phase of the cell cycle during larval development, and are poised to enter mitosis and proliferate when the rate-limiting G2-M cdc25c phosphatase String (Stg) is induced by ecdysone. Normally as a regulator of mitotic entry, Stg itself is not sufficient to drive the entire cell cycle or, therefore, proliferation (Neufeld et al., 1998). However, the dramatic proliferative response in histoblasts to the induction of Stg occurs because these cells accumulate high levels of the G1-S rate-limiting cyclin, Cyclin E (CycE), during the G2 arrest. The accumulation of CycE drives the subsequent G1-S transition, making Stg induction in these cells uniquely sufficient to induce a rapid proliferative response to remodel the abdomen during pupal stages (Ninov et al., 2009). In this manner, the pulse of ecdysone that triggers the larval-puparium transition also induces rapid and sustained cell-type specific changes in cell cycle dynamics.

In contrast to the abdominal histoblasts, other adult precursors such as the imaginal eye, wing and leg discs exhibit reduced proliferation during the early pupal stages and ultimately become post-mitotic one day after the larva-to-puparium transition (Buttitta et al., 2007; Graves and Schubiger, 1982; Milan et al., 1996; Schubiger and Palka, 1987). How can we explain the different cell cycle and survival responses of tissues to the same system-wide pulse of hormone? The differential effects of ecdysone on specific cell types has been postulated to be due in part to different ecdysone receptor (EcR) isoform expression (Cherbas et al., 2003; Davis et al., 2005; Gautam et al., 2015; Schubiger et al., 2003). The ecdysone receptor complex is a heterodimer of two nuclear receptors, EcR and Ultraspiracle (USP). Upon binding of the ecdysone ligand to EcR the EcR/USP heterodimer activates or de-represses target gene transcription. The *EcR* gene locus in *Drosophila* encodes 3 isoforms (EcR-A, EcR-B1 and EcR-B2). Each isoform has identical DNA and ligand binding domains but they differ in their N-terminal domains. In the wing, the focus of our study here, EcR-A and EcR-B1 are both expressed in the pouch which gives rise to the future wing blade, but during early metamorphosis EcR-B1 levels drop and the predominant EcR in the wing becomes the EcR-A isoform (Schubiger et al., 2003; Talbot et al., 1993). The EcR-A isoform of the receptor is thought to contain a repressive domain that is absent from the other isoforms, such that in the absence of ecdysone it represses target gene expression, but in the presence of ecdysone, these targets become de-repressed (Mouillet, 2001; Schubiger et al., 2005). In contrast to the wing, the imaginal histoblasts predominantly express EcR-B1 (Talbot et al., 1993), but this changes upon the larval-puparium transition after which histoblasts express both EcR-A and EcR-B1 isoforms (Ninov et al., 2007). While different EcR receptor isoforms may shape some of the differential responses to ecdysone in the imaginal discs versus other tissues, it is becoming clear that many targets for each receptor isoform can also be cell-type specific (Stoiber et al., 2016).

Several studies have investigated how ecdysone signaling impacts the cell cycle in larval imaginal discs. For example *ecdysoneless* (*ecd*) temperature-sensitive mutants, which severely reduce ecdysone production at the restrictive temperature, exhibit disruption of cell cycle progression in a proliferative region of the developing eye disc, termed the second mitotic wave (SMW). This wave of proliferation is preceded by the front of initial photoreceptor differentiation in the developing eye disc, termed the morphogenetic furrow, which sweeps across the disc from the posterior to anterior during late larval and early prepupal stages. In the SMW of *ecd* mutants, proliferation and expression of the mitotic cyclin, Cyclin B (CycB), is dramatically reduced (Brennan et al., 1998). Consistent with ecdysone signaling promoting proliferation, disruption of the USP component of the ecdysone receptor complex also leads to fewer proliferating cells in the area of the SMW (Zelhof et al., 1997). Ecdysone signaling has also been linked to proliferation in the larval wing imaginal disc. For example, larval wings with suppressed ecdysone signaling contain fewer and smaller cells, in part due to upregulation of the growth suppressor Thor (Herboso et al., 2015). Ecdysone signaling is also required for expression of the zinc-finger transcription factor Crooked legs (Crol), which is required in the larval wing for proper cell proliferation and survival (Mitchell et al., 2008). Furthermore, ecdysone signaling acts through Crol and Wingless to indirectly regulate CycB levels at the wing margin, an area at the dorso-ventral wing boundary where the cell proliferation pattern is distinct from the rest of the developing future wing blade (Mitchell et al., 2013). Finally, ecdysone signaling impinges on another critical growth, survival and proliferation pathway in the wing, the Hippo signaling pathway (Saucedo and Edgar, 2007). An EcR co-activator Taiman (Tai) binds to the downstream Hippo pathway transcription factor Yorkie, and is also required for normal proliferation in the larval wing pouch (Zhang et al., 2015). Thus, in the larval stages where wing cells are largely asynchronously proliferating, ecdysone signaling is required to promote proliferation and growth.

By comparison, the response of the imaginal wing disc to ecdysone during the larval-puparium transition and metamorphosis is quite different. In contrast to the asynchronous proliferation of larval wings, during metamorphosis wings undergo a series of precise temporally regulated cell cycle alterations, followed by a permanent cell cycle exit. In the prepupal wing, a temporary G2 arrest occurs at 4-6 h after puparium formation (APF). This G2 arrest is followed by a roughly synchronized final cell cycle between 12-24 h APF. Finally, the cells permanently exit the cell cycle at 24 h APF (Fain and Stevens, 1982; Milan et al., 1996; O'Keefe et al., 2012; Schubiger and Palka, 1987). A temporary G2 arrest also occurs with similar timing in the leg discs during metamorphosis (Graves and Schubiger, 1982), which subsequently undergo a final cell cycle and ultimately arrest proliferation at the same time as the wings in metamorphosis. These cell cycle alterations coincide with strong systemic pulses of ecdysone, suggesting a role for ecdysone signaling in their regulation. The temporary G2 arrest occurs as ecdysone titers drop following the pulse that triggers the larval-puparium transition, and the final cell cycle arrest occurs during the strong ecdysone pulse that triggers the onset of metamorphosis at 24 h APF (Ashburner, 1989).

A link between ecdysone signaling and the synchronized cell cycle alterations that occur in pupal wings and other appendages is consistent with observations made over 30 years ago that 20-hydroxyecdysone (HE) exposure in *Drosophila* tissue culture cells can induce a cell cycle arrest in G2 phase (reviewed in Echalier, 1997; Stevens et al., 1980). Even more provocatively, removal of 20-HE from cultured cells after a 3 day exposure, which was thought to simulate a long ecdysone pulse, triggered a transient mitotic re-entry, potentially analogous to the final cell cycle observed in imaginal discs after the G2 arrest (Besson et al., 1987). Despite thorough descriptions of these ecdysone-induced cell cycle alterations in cell culture, the mechanisms underlying the response of the cell cycle machinery to ecdysone, and how they might relate to the cell cycle changes observed *in vivo* during wing metamorphosis, have remained largely unknown. Here we reveal a pathway by which ecdysone signaling in the wing can modulate the cell cycle to coordinate cell cycle arrest with the events of cellular differentiation during metamorphosis.

## RESULTS

### 20-HE induces cell cycle arrest in G2 phase

**Fig. 1.**
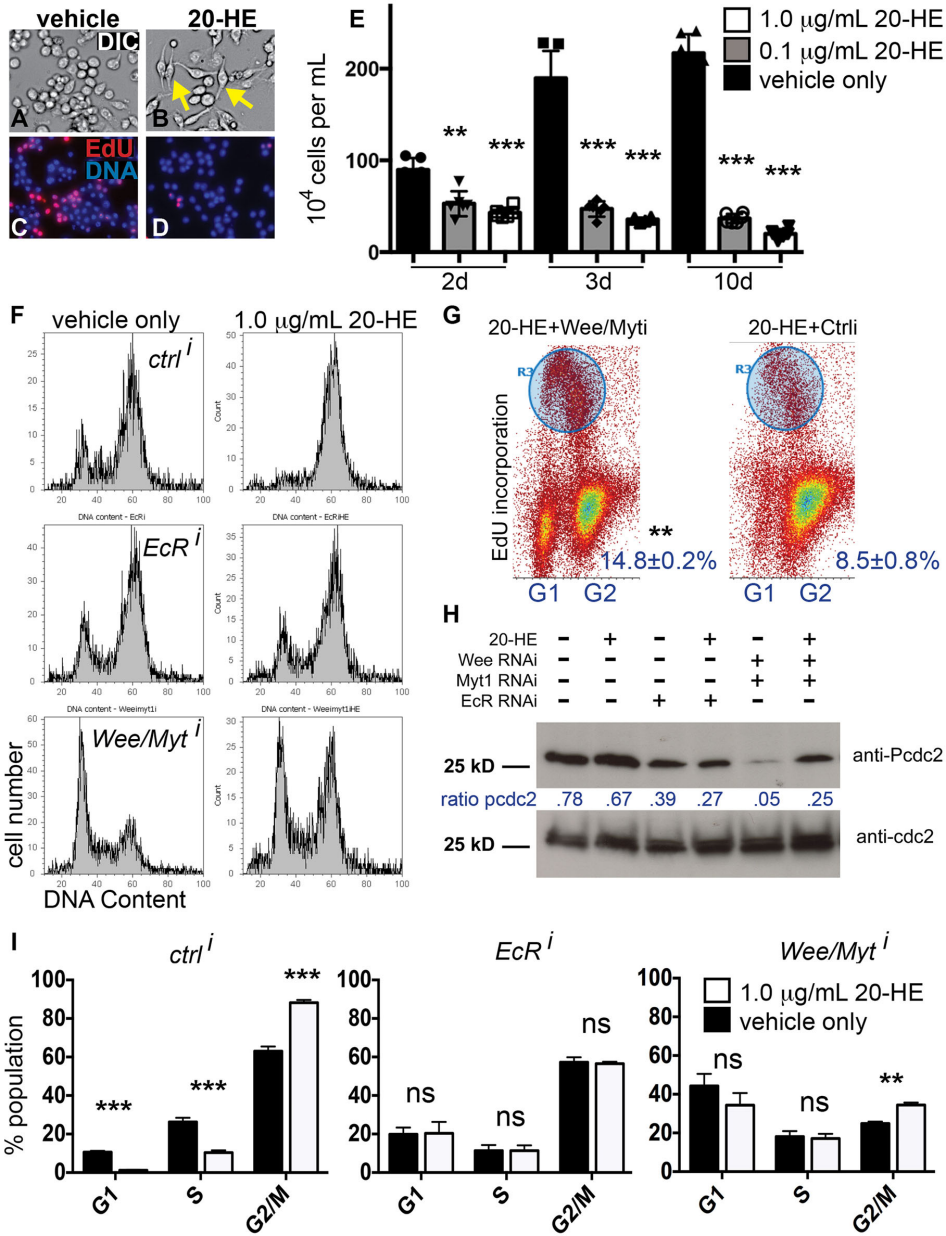
**Kc cell response to 20-HE involves a Wee/Myt-dependent cell cycle arrest in G2.** Cells were treated with vehicle only (DMSO) or 1 μg ml^−1^ 20-Hydroxyecdysone (20-HE) in DMSO for 48 h (A-I) or the indicated number of days (E). (A-D) Cells treated with 20-HE exhibit an altered cell shape and reduce EdU incorporation indicating cell cycle arrest. Yellow arrows indicate examples of the cell shape change in response to 20-HE. (E) Sustained treatment with 20-HE for 2-10 days results in fewer cells from a sustained cell cycle arrest and increased apoptosis (see also Fig. S1). Error bars indicate the s.e.m. of four replicates, *P*-values were determined by paired *t*-tests with vehicle-treated controls and range from **P<0.01 to ***P<0.001. (F) Flow cytometry confirmed that 20-HE-induced cell cycle arrest occurs in G2, which can be blocked by treatment with RNAi to the *Ecdysone Receptor* (*EcRi*) or partially blocked by RNAi to the Cdc2 kinases *wee* and *myt1* (*wee/myti*). Control (ctrl) dsRNA matches a region of Bluescript SK vector (Rogers and Rogers, 2008) and does not alter cell growth or proliferation. (G) Treatment with *wee/myt1* RNAi partially restores proliferation as shown by EdU incorporation increasing from 8% to 15% in the presence of 1 μg ml^−1^ 20-HE. (H) Levels of tyrosine-15-phosphorylated Cdc2 (pCdc2) are reduced by *EcR* RNAi and *wee/myt1* RNAis. (I) The cell cycle distribution as determined by FACS is altered in the presence of 20-HE. This effect is suppressed by knockdown of EcR. Knockdown of Wee/Myt kinases partially suppresses the increase in the G2 population in response to 20-HE. Error bars indicate the s.e.m. of four replicates, *P*-values were determined by paired *t*-tests with vehicle treated controls and range from not significant (ns=*P*>0.05) to **P<0.01 or ***P<0.001.

The levels of ecdysone signaling in specific tissues are difficult to manipulate *in vivo* without disrupting other aspects of metamorphosis. We therefore wondered whether *Drosophila* cell culture could be used as a model to examine the effects of ecdysone signaling on the cell cycle during wing metamorphosis. First, we examined the cell cycle arrest induced by 20-HE in a wing disc-derived cell line, Clone 8 (cl-8) (Peel and Milner, 1990). Cl-8 cells arrest proliferation with a G2 DNA content in response to 0.1-1 μg ml^−1^ HE treatment for 24 h (Fig. S1) (Cottam and Milner, 1997b). Unfortunately this cell line had a propensity to clump due to the secretion of cuticle proteins in response to 20-HE (Cottam and Milner, 1997a,b), which inhibited acquisition of clean, quantitative cell cycle profiles for cl-8 cells by flow cytometry. We therefore examined the cell cycle response to 20-HE in other cell lines. Treating the *Drosophila* embryonic plasmatocyte-derived cell lines Kc167 (Kc) and S2R+ with 20-HE also induced a G2 cell cycle arrest, similar to what we observe with cl-8 cells (Besson et al., 1987; Cherbas et al., 2011; Stevens et al., 1980). Kc cells undergo an easily visible cell shape change from a spherical shape to a spindle morphology in response to 20-HE within 18 h (Courgeon, 1972; Echalier, 1997), providing a convenient readout to confirm ecdysone responsiveness (Fig. 1A,B, Fig. S1). Therefore, subsequent experiments examining cell cycle responses to 20-HE in culture were performed on Kc cells. At concentrations ranging from 0.01-1 μg ml^−1^ (0.02-2.1 μM) 20-HE, a cell cycle arrest occurs in G2 phase within 18 h of exposure, reproducible in 85-95% of cells (Fig. 1C-F,I, Fig. S1). At time points after 24 h of exposure, the cell cycle arrest is sustained and accompanied by cell death (Fig. 1E, Fig. S1) (Besson et al., 1987). Prolonged exposure to 20-HE beyond 3 days selected for a small population of cells that remain rounded and resistant to the cell cycle arrest and cell death. These results are consistent with previous observations that long-term exposure selects for cells that failed to respond, or become non-responsive, to 20-HE (Besson et al., 1987; Stevens et al., 1980).

When we performed RNAi-mediated knockdown to all isoforms of the 20-HE receptor EcR, the cell cycle and cell death responses to 20-HE were completely abrogated (Fig. 1F,I Fig. S1). RNAi to EcR was highly effective (Fig. S2) and increased Kc cell proliferation and survival, even in the absence of exogenously provided 20-HE, suggesting that under normal culture conditions Kc cells respond to low levels of endogenous 20-HE (Fig. S1). This may be responsible for the increased G2 population we and others have observed in Kc versus S2R+ cells (Wu et al., 2007).

### The Wee/Myt1 kinases are partially responsible for 20-HE induced G2 arrest

To determine how 20-HE blocks the cell cycle in G2, we performed RNAi treatments to knock down key cell cycle regulators of G2 phase and exposed cells to 1 μg ml^−1^ 20-HE for 24-48 h. We then examined cell cycle phasing by FACS (Fig. 1F) and quantified cell cycle arrest by EdU incorporation (Fig. 1G). A non-specific dsRNA fragment from the Bluescript SK vector, previously shown not to affect cell viability or cycling, served as our control RNAi (Rogers and Rogers, 2008). Knocking down the Cdc2 inhibitory kinases Wee and Myt1 together effectively bypassed the 20-HE induced G2 arrest in about half of the population (Fig. 1F,G), but did not fully suppress the 20-HE induced increase in G2 cells (Fig. 1I, *P*<0.001). Knockdown of Wee and Myt1 was effective and also affected the cell cycle in cells exposed to vehicle only, leading to a substantially increased proportion of G1 cells compared to control RNAi (Fig. 1F,I, Fig. S2) and increased cell proliferation (data not shown).

We next examined the phosphorylation state of Cdc2 using an antibody recognizing the inhibitory phosphorylation catalyzed by Wee and Myt1 (anti-pCdc2). The knockdown of Wee and Myt1 was effective, as it substantially reduced Cdc2 phosphorylation in unexposed Kc cells, which already exhibit a significant fraction of cells in G2-phase (Fig. 1H, Fig. S2). 20-HE exposure together with Wee/Myt RNAi led to an increase in p-Cdc2 compared to treatment with the RNAi alone, consistent with our FACS showing that the G2 arrest induced by 20-HE partially persists even when Wee/Myt1 are strongly reduced (Fig. 1F,I). These data indicate that the 20-HE induced cell cycle arrest is mediated at least in part by phosphorylation of Cdc2 by Wee and Myt1.

### The 20-HE induced G2 arrest is reversible

**Fig. 2.**
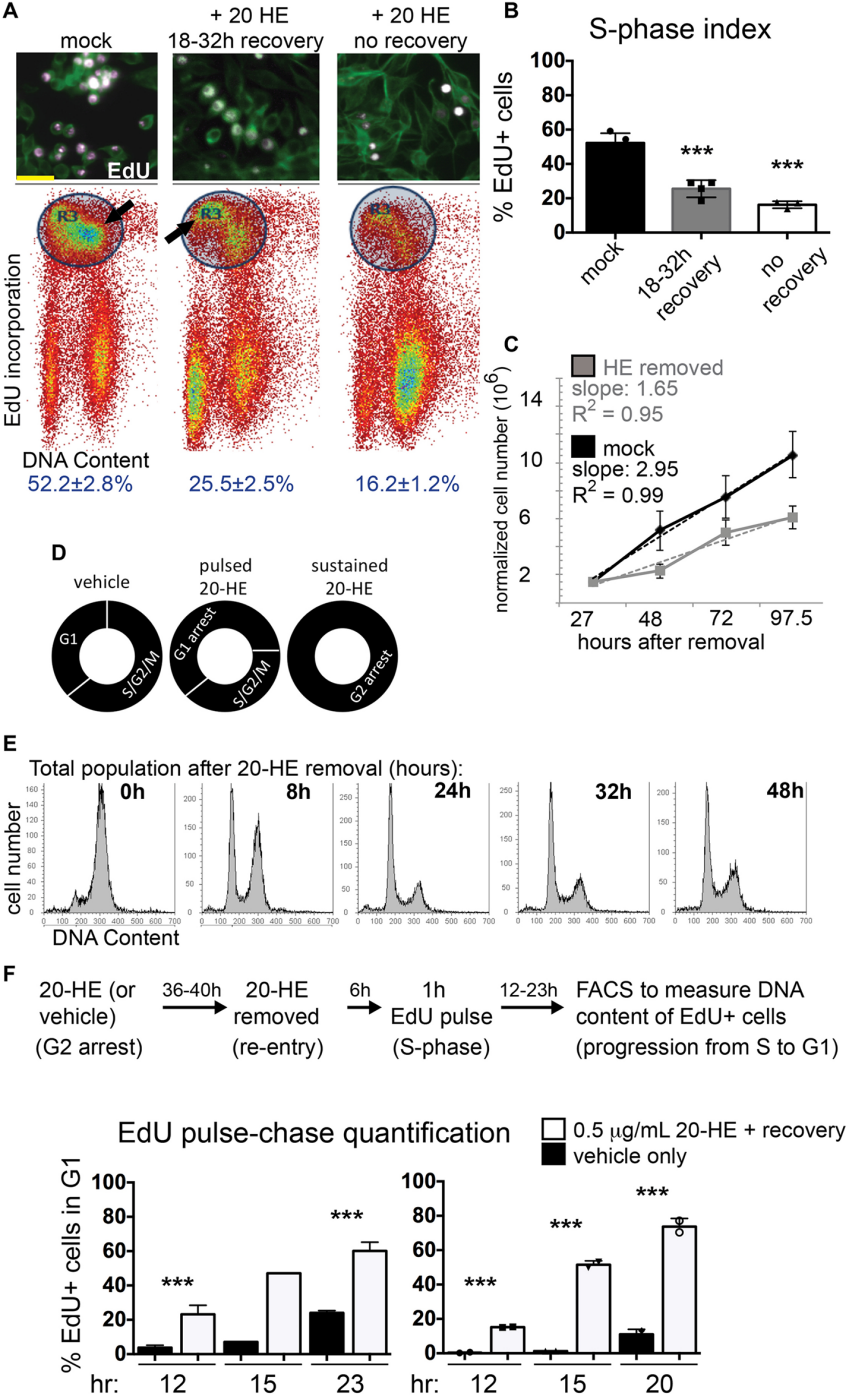
**20-HE**-**induced arrest is reversible and leads to prolonged alterations in cell cycle dynamics.** (A) Cells were treated with vehicle (mock) or 1 μg ml^−1^ 20-HE for 48 h followed by removal and replacement with fresh media for the indicated number of hours; ‘no recovery’ indicates continued incubation with 20-HE. EdU incorporation for 45 min was used to examine cell cycle re-entry after 20-HE removal. Lower panels show flow cytometry density plots of cells labeled for EdU and DNA content with colors indicating the density or relative number of cells in a certain region. Gating (indicated by the blue circle) was used to identify EdU positive early and late S-phase cells (indicated by arrows). (B) Quantification of EdU-positive cells, error bars indicate s.e.m. of four replicates, *P*-values were determined by *t*-test compared to mock treated controls, ***P<0.001. Removal of HE for 18-32 h only partially restores EdU incorporation. (C) Normalized cell counts after mock treatment or removal of 20-HE for the indicated hours. Cells treated with 20-HE fail to recover normal proliferation even several days after hormone removal. Error bars indicate s.e.m. of four replicates. (D) A diagram depicting the relative cell cycle phasing of Kc cells with the indicated treatments. (E) Cells were treated with 0.5 μg ml^−1^ 20-HE for 36 h followed by removal, washing and media replacement for the indicated number of hours. One to two days after 20-HE removal the majority of cells exhibit a G1 DNA content, suggesting a rapid cell cycle re-entry followed by a G1 arrest. For quantification see Fig. S2A. (F) An EdU pulse-chase experiment was performed to track the cell cycle progression of cells after 20-HE removal. Cells that re-enter the cell cycle 6 h after 20-HE removal were pulsed with EdU and followed for the indicated number of hours. The percentage of EdU-positive cells that progress from S-phase through the cell cycle to G1 after 20-HE removal is shown. S to G1 progression proceeds more rapidly after 20-HE exposure. Two independent pulse-chase experiments with two replicates are shown. Paired *t*-tests indicate significant differences (***P<0.001) between mock treated controls and 20-HE removal.

Previous work mapping origins of replication in Kc cells used a protocol of 20-HE-induced G2 arrest followed by release into media without 20-HE, containing hydroxyurea to synchronize cells in S-phase (MacAlpine et al., 2004). This suggested the 20-HE-induced G2 arrest was reversible upon 20-HE removal for at least a proportion of cells. To examine how the exposure to a pulse of 20-HE affected subsequent cell cycles following 20-HE removal, we performed a 20-HE G2 arrest and release protocol with EdU labeling, and then we measured the fraction of cells that re-enter the cell cycle following G2 arrest by FACS analysis. We exposed cells to 1 μg ml^−1^ 20-HE for 24 h and removed the 20-HE by performing washes and providing fresh media. After allowing cells to recover for the indicated times, we counted cells to measure their proliferation rate or exposed them to EdU for a duration sufficient to label about 50% of the total population to measure S-phase re-entry (Fig. 2A,B). Since the addition of fresh media alone can alter cell cycle dynamics in culture, we treated mock controls the same way, in parallel using vehicle only.

We found that 18-32 h after recovery from 20-HE-induced cell cycle arrest, about 26% of the cells have completed mitosis and re-entered the cell cycle progressing into a subsequent S-phase (Fig. 2A,B). This is in comparison to over 50% of the mock control, which suggests that roughly one half of cells can re-enter a subsequent cell cycle following a 20-HE-induced G2 arrest and 20-HE removal. Cells exposed to 20-HE continually for 46 h also exhibit a low level of EdU incorporation (about 16%), which may be due to a combination of prolonged cell death and arrest over 2 days, selecting for a population of cells that failed to arrest initially or cells that lose responsiveness to 20-HE (Fig. 2A,B). Thus, a transient pulse of 20-HE initiates a temporary G2 arrest, followed by re-entry into the cell cycle after 20-HE removal.

### 20-HE removal leads to prolonged alterations in cell cycle dynamics

The proliferation rate of cells after a 20-HE-induced arrest also remains slower than that of mock treated controls, even up to 97 h after 20-HE removal (Fig. 2C). This could be due to persistent cell death that continues in response to the 20-HE even after removal (Besson et al., 1987), or an alteration of the subsequent cell cycle, such as a prolonged G1 or G1 arrest (Fig. 2D). To distinguish between these two possibilities, we performed a time course analysis of the cell cycle phase after 20-HE removal. We performed this time course under conditions that reduce the amount of apoptosis in response to 20-HE, by reducing the concentration of 20-HE to 0.5 μg ml^−1^. Under these conditions, as quickly as 8 h after 20-HE removal, a substantial fraction of cells have re-entered the cell cycle and proceed through mitosis and into the subsequent G1 phase (Fig. 2E). By 24 h after 20-HE removal many cells are in G1 phase (42%). By 32-48 h after 20-HE removal we begin to see an increase in cycling cells in S and G2 phases, but the majority of the population remains in G1 (55%), suggesting a substantially prolonged G1 or a subsequent G1 arrest in a significant portion of the population occurs following 20-HE removal (Fig. 2E, quantification in Fig. S2A).

We were surprised to find that few cells were in S or G2 phase 24 h after 20-HE removal, considering that our EdU labeling experiment indicated that about half of cells can re-enter the cell cycle and proceed through an S-phase after 20-HE removal (Fig. 2A,B). This suggests that a prolonged G1 or G1 arrest may follow cell cycle re-entry after 20-HE removal. To test this hypothesis we performed an EdU pulse chase experiment to follow the cell cycle timing of cells that re-enter S-phase after a pulse of 20-HE. We exposed cells to 20-HE to 0.5 μg ml^−1^ for 36-40 h followed by removal. Cells were allowed to recover for 6 h, then pulsed with EdU for an hour followed by a chase without EdU or 20-HE for the indicated times. We then assayed the EdU-labeled cells by FACS, starting 4 h after the EdU pulse, which is equivalent to 12 h after 20-HE removal. At this time, 15.2-23.2% of EdU-labeled cells have progressed from S-phase through mitosis to the subsequent G1 phase. This is in contrast to 3.8% of the mock-treated control, indicating that after 20-HE release the subsequent progression from S-phase to the next G1 proceeds much more rapidly than in mock-treated controls. By 20-23 h after 20-HE removal (13-16 h post EdU labeling) 60.1-74.0% of 20-HE-pulsed cells have completed mitosis and now exhibit a G1 DNA content, while only 24% have done so in the mock-treated control. Altogether this suggests that after the release from a 20-HE-induced G2 arrest, cells that re-enter the cell cycle exhibit a rapid S/G2/M progression, followed by a subsequent prolonged G1 phase (Fig. 2D). This is consistent with the large G1 population we observe 24 and 32 h after 20-HE removal in Fig. 2E.

### Cell cycle changes during wing metamorphosis are similar to those induced by pulsed 20-HE in cell culture

**Fig. 3.**
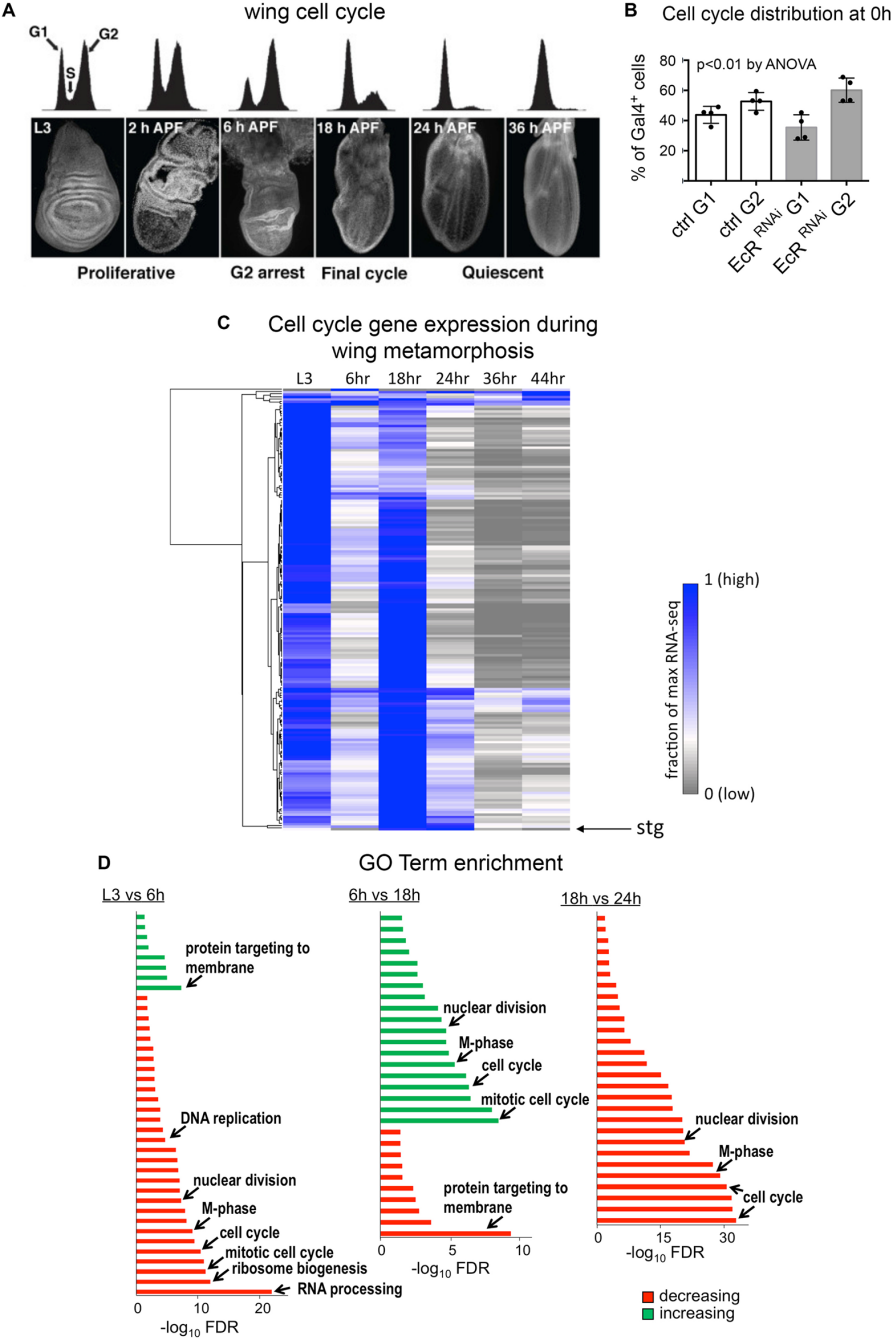
**Cell cycle changes during wing metamorphosis.** (A) *Drosophila* wings undergo a temporary G2 arrest at 6 h after puparium formation (APF) followed by one additional cell cycle and a subsequent G1 arrest as indicated by flow cytometry on staged, dissected wing tissues. This figure is reproduced from (O'Keefe et al., 2012) and is also shown in Fig. 7. (B) RNAi to all isoforms of EcR was expressed in the dorsal compartment of larval wings using *apterous-Gal4*, *UAS*-*GFP*, *Gal80^TS^* (*Gal4+*). EcR RNAi+GFP or GFP alone (ctrl) was expressed for 24-96 h prior to dissection, dissociation and analysis by FACS at 0 h APF for cell cycle distribution in G1 and G2. Inhibition of EcR resulted in a significant fraction of cells at 0 h APF exhibiting a G2 arrest. Error bars indicate the standard deviation of four replicates. (C) RNAseq was performed on dissected wings at the indicated timepoints to monitor changes in gene expression. A core cluster of 183 cell cycle genes exhibit dynamic regulation during wing metamorphosis, with high expression during the proliferative L3 stage and the final cell cycle, followed by very low expression after cell cycle exit at 24 h. Most cell cycle genes also decrease expression during the G2 arrest at 6 h, although *string* (*stg*) behaves as an outlier in this cluster, showing the most dramatic decrease at 6 h. (D) Analysis of GO term enrichment revealed that genes involved in cell cycle and cell growth (ribosome biogenesis) decrease during the G2 arrest at 6 h. During cell cycle re-entry (18 h) cell cycle genes are upregulated to promote progression through a rapid final cell cycle. Cell cycle genes are again strongly downregulated at 24 h to promote cell cycle exit.

The pattern of cell cycle changes we observed in cell culture in response to a pulse of 20-HE is highly reminiscent of the cell cycle dynamics in the *Drosophila* wing blade during early pupal stages (Fig. 3A, O'Keefe et al., 2012). The wing blade transitions from a largely asynchronously proliferating tissue in the late third instar larval stage (L3), to a mostly G2-arrested tissue at 6 h after the larval-puparium transition (hours after puparium formation or h APF). After the G2 arrest in the wing, a fraction of cells re-enter the cell cycle at 12-18 h APF and progress through an additional cell cycle followed by a sustained G1 arrest as the wing becomes quiescent and begins terminal differentiation (Milan et al., 1996; O'Keefe et al., 2012; Schubiger and Palka, 1987). Since the larval-puparium transition is triggered by a pulse of ecdysone (Ashburner, 1989), we wondered whether the temporary G2 arrest at 6 h in the wing may be caused by ecdysone signaling at 0 h APF, similar to what we observed in cell culture. The EcR-A/USP complex, the predominant ecdysone receptor complex in the wing, has been shown to repress target genes in the absence of ecdysone, and binding of ecdysone to EcR-A is thought to relieve this repression. Therefore, we reasoned that knockdown of all EcR isoforms early in wing development could be used to mimic a precocious ecdysone exposure at 0 h APF, similar to previous findings (Ghbeish et al., 2001; Schubiger and Truman, 2000). To determine whether the G2 arrest in the early pupal wing is due to ecdysone signaling, we knocked down all EcR isoforms by expressing an EcR RNAi in the dorsal wing using *apterous-Gal4*, *UAS*-*GFP*, *Gal80^TS^* (*ap-Gal4*) for 24-96 h and collected wings at 0 h APF. We examined the cell cycle distribution in wings by FACS to determine the relative percentages of cells in G1 and G2 compared to controls without the EcR RNAi. Consistent with the G2 arrest we observe in cell culture, we find that knockdown of EcR in the wing resulted in a significant fraction of cells at 0 h APF exhibiting a precocious G2 arrest (Fig. 3B). This suggests that ecdysone signaling is sufficient to induce a G2 arrest in the larval *Drosophila* wing.

We next performed an RNAseq time course on wings from L3 to 44 h APF to examine the global changes in gene expression during metamorphosis. These timepoints include the L3 larval stage (−10 h APF), the temporary G2 arrest (6 h APF), the final cell cycle (18 h APF), the permanent cell cycle arrest (24 h APF), and stages of terminal differentiation including vein differentiation, wing hair formation and cuticle protein production (36 and 44 h APF). We observed a striking number of gene expression changes during this time course, with over 2000 genes (∼12% of the genome) significantly changing expression in the wing within the first 6 h of metamorphosis (Fig. S3). Subsequent timepoints also showed dramatic changes in gene expression ranging from over 1900 genes changing between 6 h and 18 h APF during cell cycle re-entry from the G2 arrest, to nearly 650 changing between 18 and 24 h APF when cells complete the final cell cycle and enter quiescence (Fig. S3).

We examined a set of 183 core cell cycle genes that include the cell cycle Cyclins/Cdks and their key targets and regulators (Fig. 3C, Table S1). Cell cycle genes showed generally similar behavior during wing metamorphosis with high expression in the proliferating L3 wing and during the final cell cycle at 18 h APF, but lower expression during the 6 h G2 arrest and very low expression after completion of the final cell cycle at 24 and 36 h APF. We observed reduced mRNA expression for many G2/M cell cycle regulators during the G2 arrest at 6 h APF including the mitotic *aurora-A* and *polo* kinases, the G2/M cyclin *cyclinB* and the mitotic checkpoint regulators *mad2*, *bub3* and *fizzy*, the *Drosophila* homolog of cdc20 (Table S1). However, we noted that the gene showing the most dramatic reduction specifically at 6 h APF was the rate-limiting G2/M phosphatase *string* (*stg*), which removes the phosphorylation on cdc2 catalyzed by the Wee/Myt kinases (Fig. 3C).

We next examined the global gene expression changes during wing metamorphosis for enrichment of specific gene ontology (GO) terms. Consistent with the changes in cell cycle genes we observed, we found that cell cycle-associated terms such as ‘M-phase’, ‘nuclear division’ and ‘mitotic cell cycle’ were greatly enriched in the genes that decrease expression at 6 h APF (Fig. 3D, Fig. S4). Conversely, the genes that increase expression between 6 and 18 h APF during the final cell cycle are strongly enriched for this same set of cell cycle terms, which decrease expression again during cell cycle exit at 24 h APF.

There is also a strong enrichment for genes involved in ‘RNA processing’ (including rRNA) and ‘ribosome biogenesis’ in the genes that decrease expression at 6 h APF (Fig. 3D). This is consistent with the observation that the final two cell cycles in the *Drosophila* wing are reductive divisions that occur in the absence of significant cellular growth, resulting in smaller cells which ultimately become stretched and flattened to increase apical area later during wing elongation (our unpublished observations; Aigouy et al., 2010, O'Keefe et al., 2012). Altogether our GO term analysis suggests that a significant fraction of the dramatic gene expression changes we observe in the wing during metamorphosis are driven by changes in the cell cycle dynamics of the pupal wing.

### A peak of Broad-Z1 expression is correlated with the G2 arrest in the wing

**Fig. 4.**
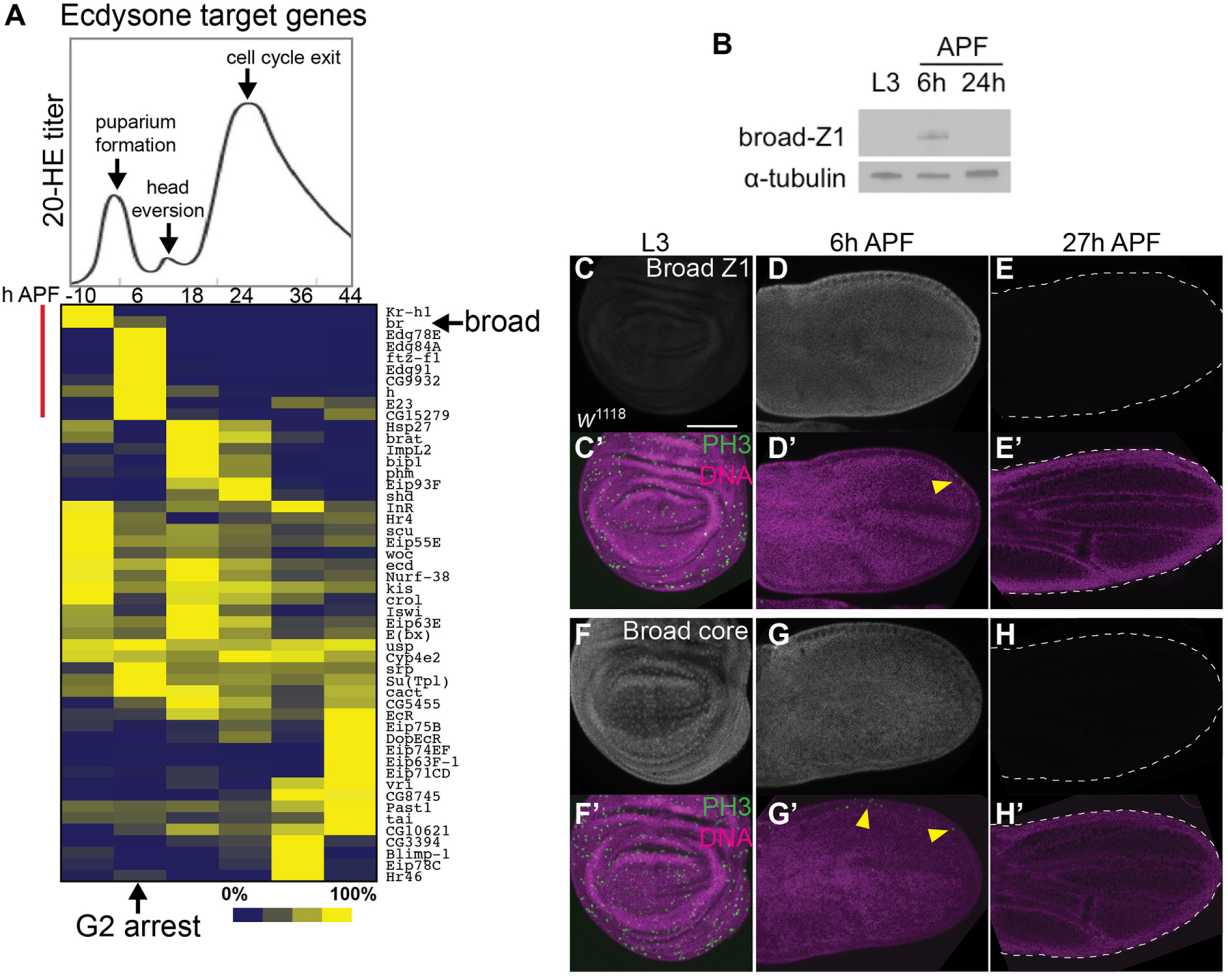
**G2 arrest in the prepupal wing coincides with a peak of Broad Z1 expression.** (A) The titer of ecdysone in animals from −10 h APF to 44 h APF (at 25°C) is shown. This graph is adapted from (Ashburner, 1989). Known ecdysone targets were clustered using Pearson correlation coefficients according to their expression changes during metamorphosis in the wing by RNAseq. Each gene is represented as a fraction of its maximum expression across the time course. The top cluster (indicated by a red line) contains targets induced specifically prior to or during the G2 arrest. The RNAseq heatmap signal for the direct ecdysone target *broad* encompasses all isoforms. (B) Endogenous BroadZ1 (BrZ1) protein levels were assayed in dissected wings of the indicated stages by western blot. BrZ1 peaks at 6 h. Alpha-tubulin from the same blot serves as a protein loading control. (C-H) Wings of the indicated stages were dissected, fixed and immunostained for all Broad isoforms (Broad core) or BrZ1. Broad isoforms are expressed at L3 stage prior to puparium formation, while BrZ1 is specific to the early prepupal wing. All images were taken with identical gain and laser intensities for comparison. Yellow arrowheads indicate mitoses that occur at the wing anterior margin at 6h APF. Scale bar: 100 µm.

From our RNAseq data we next extracted a set of known ecdysone signaling targets (Beckstead et al., 2005; Gauhar et al., 2009; Shlyueva et al., 2014) and plotted their expression levels over time in a clustered heat map, superimposed with the ecdysone titer and key events during these pupal timepoints (Fig. 4A, 20-HE titer graph reproduced from Ashburner, 1989). This revealed multiple waves of ecdysone responses in the pupal wing, including early and late responses induced by the larval-prepupal pulse, as well as distinct early and late responses to the larger pulse at 24 h APF. We noted a cluster of direct targets of ecdysone that are expressed prior to or during the G2 arrest at 6 h APF but are not induced by the second ecdysone pulse at 24 h (Fig. 4A, cluster indicated by red line). This cluster included 5 transcription factors, *Kr*-*h1*, *broad*, *ftz-f1*, *hairy (h)* and *CG9932*. We decided to examine Broad further as a potential regulator for the G2 arrest in the early pupa wing, since the knockdown of EcR by RNAi that induced a precocious G2 arrest in the wing (Fig. 3B) was previously shown to induce precocious expression of a specific isoform of Broad, Broad-Z1 (Schubiger and Truman, 2000). Importantly, we also found Broad-Z1 to be specifically enriched in wings at 6 h APF by western blot (Fig. 4B) and immunofluorescence (Fig. 4C-E), coincident with the G2 arrest. We also detected total Broad expression in wings using an antibody to the Broad-core region that recognizes all Broad isoforms. We found that other isoforms of Broad, most likely transcripts encoding the Z3 isoform (based on our RNAseq data), are expressed in the larval wing prior to the larval-puparium transition. However, we saw no Broad expression at 27 h APF following the largest ecdysone pulse, suggesting ecdysone only induces Broad-Z1 in the early prepupal wing (Fig. 4F-H). Thus, Broad expression, particularly the Z1 isoform, coincides with the ecdysone-induced G2 arrest in early pupal wings.

### Broad regulates the ecdysone-induced G2 cell cycle arrest in early pupal wings

**Fig. 5.**
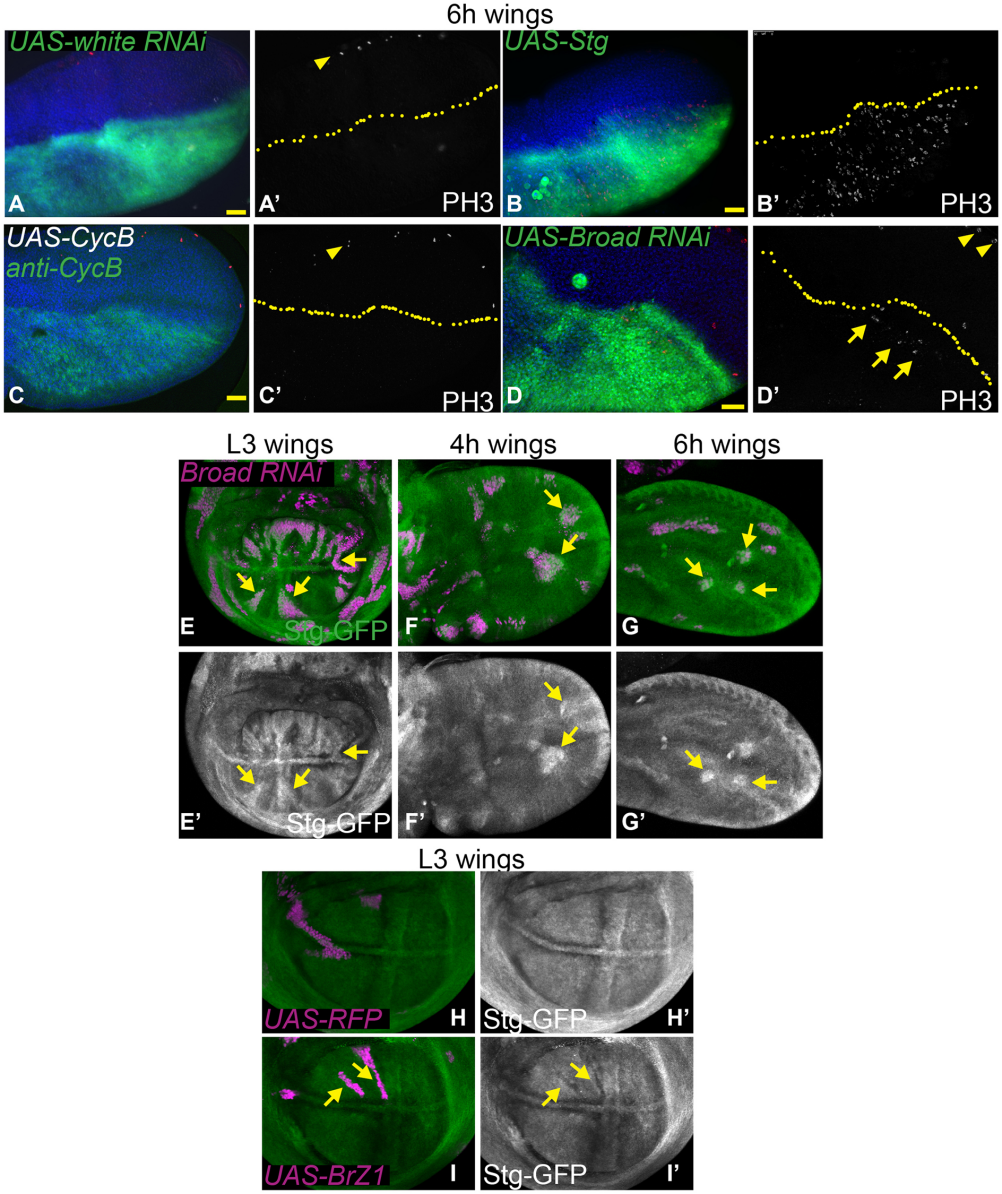
**The prepupal wing G2 arrest is partially dependent upon Broad regulation of String.** (A-D) *en-gal4* coupled with a temperature-sensitive *tubulin-Gal80*(*TS*) was used to limit UAS-driven expression from mid-L3–6 h APF to avoid defects in pupariation. Wings were dissected at 6 h APF and stained for mitoses using anti-phosphohistone H3 (PH3) (Su et al., 1998). (A,A′) A UAS transgene driving expression of RNAi to the eye pigment gene *white* was used as a negative control. At 6 h APF mitoses are normally restricted to the anterior margin (arrowhead) but are absent from the wing blade (*n*=6/6). (B,B′) Expression of *string* in the posterior wing bypasses the G2 arrest and drives cells of the posterior wing into an early mitosis (*n*=7/7), (C,C′) while overexpression of CycB does not (*n*=8/8). For A-D, yellow arrowheads indicate mitoses that occur at the wing anterior margin at 6h APF. (D,D′) Knockdown of *Broad* leads to ectopic mitoses in the posterior wing (yellow arrows, *n*=9/14). For A-D, yellow arrowheads indicate mitoses that occur at the wing anterior margin at 6h APF. (E-I) A Stg-GFP protein trap was recombined with an *actin-“flipout stop”-Gal4* transgene (*act>Gal4*) to generate heat-shock induced *flp/FRT, UAS-RFP* labeled clones expressing the indicated UAS transgenes. (E-G) *Broad* RNAi increases Stg-GFP at L3, 4 and 6 h APF (H-I) while BrZ1 overexpression decreases Stg-GFP levels. Representative experiments are shown, which were independently replicated 2-3 times. For E-I, yellow arrows indicate clones exhibiting altered Stg-GFP expression. Scale bar: 25 µm.

We have shown that the 20-HE–induced G2 arrest in cell culture relies in part upon the Wee/Myt kinases, which phosphorylate and inhibit Cdc2 activity (Fig. 1). We also found that *stg* expression, which counteracts the activity of the Wee/Myt kinases on Cdc2, is very low at 6 h APF during an ecdysone-induced G2 arrest in the wing. Therefore, we tested whether the G2 arrest in the early prepupal wing was dependent upon inhibitory Cdc2 phosphorylation. To do this, we used the *engrailed*-*Gal4* driver with a temperature sensitive Gal80 (*en-Gal4/Gal80^TS^*) to turn on ectopic expression of UAS-driven *stg* and GFP in the posterior wing at 0 h APF. We then dissected wings at 6 h APF and stained for the mitotic marker phosphorylated Ser-10 histone H3 (PH3) to determine whether cells in the posterior wing bypassed the G2 arrest and entered into mitosis. Indeed, ectopic expression of *stg* robustly bypassed the G2 arrest in 100% of wings at this stage, demonstrating that the arrest is dependent upon inhibitory phosphorylation of Cdc2 (Fig. 5A,B). Previous work had shown that ecdysone signaling could regulate Cyclin B (CycB) levels in the larval wing margin indirectly through another ecdysone-regulated transcription factor *crooked legs* (*crol*) (Mitchell et al., 2013). Since CycB also regulates Cdc2 activity and *cyclin B* levels were also low at 6 h APF in our RNAseq data, we tested whether overexpression of CycB could bypass the G2 arrest. However, in 100% of wings robust expression of CycB failed to promote entry into mitosis (Fig. 5C).

We next asked whether inhibition of Broad altered the G2 arrest in the early pupal wing. To inhibit Broad we used a UAS-driven RNAi that targets a common region of *broad* transcripts effectively knocking down all isoforms with the *en-Gal4/Gal80^TS^* driver (Fig. S2). Expression of the *broad* RNAi from the early second larval instar disrupted pupariation and resulted in visible defects in pupa cuticle tanning similar to those reported for overexpression of the repressive EcR-A isoform (Fig. S2, Schubiger et al., 2003). By contrast, expression of the RNAi from 0 h APF did not show any visible effects on pupal development, presumably due to insufficient knockdown. Therefore, we used *Gal4/Gal80^TS^* to drive Broad RNAi expression for only 18-24 h prior to 6 h APF, which likely results in a partial knockdown. Nevertheless, expression of *broad* RNAi at these moderate levels resulted in ectopic mitoses in the posterior wing of 75% of animals (ranging from 3-14 mitoses/wing, Fig. 5D), consistent with a role for Broad in promoting the G2 cell cycle arrest.

To determine whether Broad may regulate Stg levels, we used a Stg-GFP protein trap line to monitor endogenous Stg expression (Inaba et al., 2011). We combined this protein trap with an *actin-“flipout stop”-Gal4* transgene (*act>Gal4*) to generate heat-shock induced *flp/FRT, UAS-RFP*-labeled clones expressing Broad RNAi or a control RNAi to the *white* gene that has no effect on the cell cycle. Clones expressing Broad RNAi exhibit increased expression of Stg-GFP from L3 to 6 h APF in the wing (Fig. 5E-G, arrows; Fig. S2). Conversely, ectopic Broad-Z1 overexpression reduces Stg-GFP expression in the proliferating L3 wing (Fig. 5H-I, Fig. S2). Altogether our data suggests ecdysone signaling acts via Broad to downregulate String to induce a temporary G2 arrest in the pupa wing. The G2 arrest in the early pupal wing can be bypassed by reducing Broad or by providing exogenous String.

### Broad binds to the *stg* regulatory locus and overlaps with wing enhancers

**Fig. 6.**
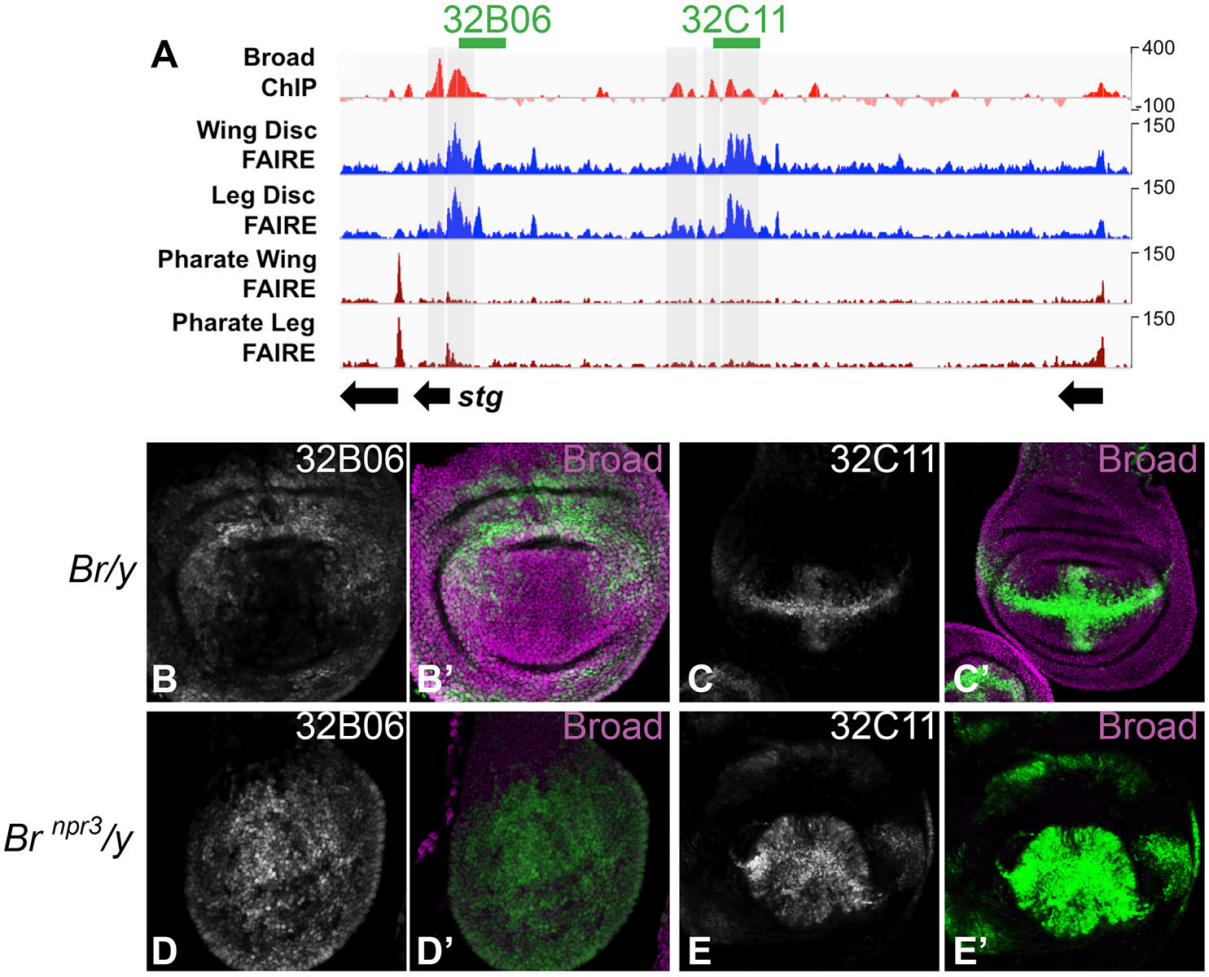
**Broad binds to the *stg* regulatory locus and overlaps with wing enhancers.** (A) An 80 kb window of the *stg* locus is shown including Broad ChIP-seq signal at 0 h APF (modencode dataset #3806, www.modencode.org), and potential regulatory elements revealed by FAIRE that are accessible during larval stages in the wing and leg disc, but become inaccessible after cell cycle exit in the pharate tissues (McKay and Lieb, 2013). Two regions are shown that overlap with reporters from the Janelia Gal4 collection previously shown to drive expression in the wing (Andrade-Zapata and Baonza, 2014). (B-C) Male animals hemizygous for *broad* show expression of these reporters in the wing. (D-E) Animals hemizygous for the *broad npr3* null allele show loss of Broad expression and ectopic reporter expression in the central wing pouch (for 32B06) and additional expression outside of the anterior-posterior and dorsal-ventral boundary regions (for 32C11). GFP expression is shown in green, and Broad protein expression is shown in magenta.

To investigate whether Broad may regulate Stg expression directly, we examined Broad binding to the *string* gene locus at 0 h APF using modencode dataset #3806 (www.modencode.org). The *string* regulatory region is modular and extensive, spanning >40 kb (Lehman et al., 1999; Lopes and Casares, 2015), and several regulatory elements that drive expression in the wing have been previously described (Andrade-Zapata and Baonza, 2014). These regions overlap with accessible potential regulatory elements identified by formaldehyde-assisted isolation of regulatory elements (FAIRE) that become inaccessible after cell cycle exit in the late pharate adult tissues (McKay and Lieb, 2013). By aligning Broad binding data with FAIRE data at the *stg* locus, we found that several strong peaks of Broad binding overlap with potential wing regulatory elements (Fig. 6A).

Two Gal4 reporters generated from *stg* regulatory regions (from the Janelia Gal4 collection) that drive expression in the wing overlap with peaks of Broad binding, 32B06 and 32C11 (Andrade-Zapata and Baonza, 2014). We therefore examined whether the loss of Broad alters the expression of these reporters. The *broad* locus is located on the X-chromosome, in males hemizygous for wild-type *broad* we observed normal Broad protein levels and the previously described expression patterns for these reporters in the wing (Fig. 6B,C). By contrast, in animals hemizygous for the *broad npr3* null allele, Broad expression is lost and ectopic reporter expression is observed in the central wing pouch for 32B06, and additional expression in the wing pouch and hinge is observed for 32C11 (Fig. 6D-E). Loss of Broad also disrupts larval development and wing patterning (Jia et al., 2016), resulting in reduced wing size and altered wing pouch shape. Altogether these data demonstrate that Broad binds *stg* regulatory regions that drive expression in the wing. Moreover, they suggest that Broad (most likely the Z1 isoform) induces the G2 arrest at 6 h APF by directly repressing *stg* expression.

## DISCUSSION

**Fig. 7.**
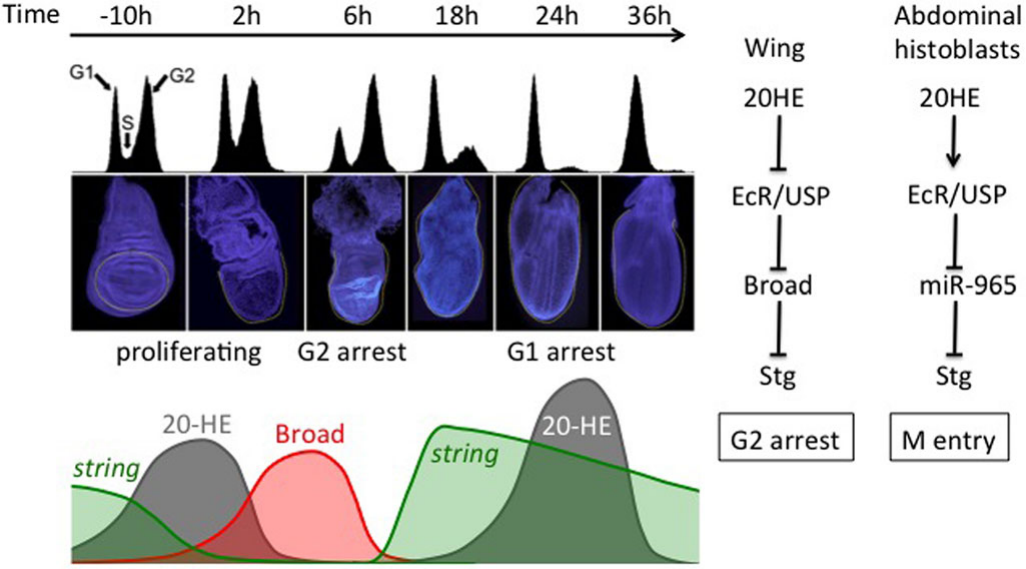
**A model for how the temporal dynamics of Ecdysone signaling induce two phases of cell cycle arrest in *Drosophila* wings.** Pulses of Ecdysone signaling, Broad expression and String expression are temporally regulated during wing metamorphosis to coordinate cell cycle changes with differentiation and morphogenesis. In the wing, ecdysone relieves EcR/USP repression of Broad, which in turn regulates Stg. The outcome is opposite to that of the recently worked out molecular pathway for G2 progression in the abdominal histoblasts (Verma and Cohen, 2015). A portion of this figure, showing cell cycle changes in the wing during metamorphosis, is reproduced from (O'Keefe et al., 2012) and also shown in Fig. 3.

We present a model for how the pulse of ecdysone at the larval-to-pupal transition impacts the cell cycle dynamics in the wing during metamorphosis (Fig. 7). Ecdysone signaling at the larva-to-puparium transition induces Broad, which in turn represses Stg to generate a temporary G2 arrest, which synchronizes the cell cycle in the wing epithelium. As ecdysone levels decline, Broad expression plummets, allowing Stg to be re-activated resulting in a pulse of cdc2 activity that promotes a rapid G2/M progression during the final cell cycle in the wing. This ultimately culminates in the relatively synchronized cell cycle exit at 24 h APF (Milan et al., 1996; Schubiger and Palka, 1987), coinciding with the second large pulse of ecdysone. This second pulse in the pupa activates a different set of transcription factors (not Broad), promoting the acquisition of terminal differentiation characteristics in the wing. In this way, two pulses of ecdysone signaling can both synchronize the final cell cycle by a temporary G2 arrest and coordinate permanent cell cycle exit with the acquisition of terminal differentiation characteristics in the wing.

Over 30 years ago it was shown that 20-HE exposure in *Drosophila* tissue culture cells induces a cell cycle arrest in G2-phase (reviewed in Echalier, 1997; Stevens et al., 1980). This response appears to be shared among three different cell lines, Cl-8, Kc and S2 (Fig. S1). Here, we show that in Kc cells pulsed 20-HE exposure also leads to a G2 arrest followed by rapid cell cycle re-entry after 20-HE removal and a subsequent prolonged G1 (Fig. 2). This cell cycle response to a pulse of 20-HE is reminiscent of the cell cycle changes that occur during early metamorphosis in the pupal wings and legs (Graves and Schubiger, 1982; O'Keefe et al., 2012).

It is worth considering why Kc and S2 cells, which are thought to be derived from embryonic hemocytes, would exhibit a similar cell cycle response to 20-HE to the imaginal discs. Relatively little is known about how ecdysone signaling impacts embryonic hemocytes, although recent work suggests that ecdysone signaling induces embryonic hemocyte cell death under sensitized conditions (Sopko et al., 2015). More is known about larval hemocytes, which differentiate into phagocytic macrophages and disperse into the hemolymph during the first 8 h of metamorphosis (Grigorian et al., 2011). Ecdysone is involved in this maturation process, as lymph glands of *ecdysoneless* (*ecd*) mutants fail to disperse mature hemocytes and become hypertrophic in the developmentally arrested mutants (Sorrentino et al., 2002). This suggests that the high levels of systemic ecdysone signaling at the larval-puparium transition mediate a switch from proliferation to cell cycle arrest and terminal differentiation for lymph gland hemocytes during metamorphosis. Without ecdysone signaling, hemocytes may continue to proliferate and fail to undergo terminal differentiation leading to the hypertrophic lymph gland phenotype observed. Interestingly, while the loss of *broad* also prevents proper differentiation of hemocytes similar to loss of *ecd*, loss of *broad* does not lead to the hypertrophy observed in *ecd* mutants (Sorrentino et al., 2002). Further studies will be needed to examine whether the ecdysone induced cell cycle arrest in larval hemocytes occurs in the G2 phase, or whether their cell cycle arrest proceeds via a similar pathway to that shown here for the wing.

### The ecdysone receptor is a repressive complex in the wing

Multiple lines of evidence suggest that the ecdysone receptor complex in the larval wing acts as a repressor for certain early pupa targets and that the binding of ecdysone to the receptor relieves this repression (Fig. 7). For example loss of EcR by RNAi or loss of the EcR dimerization partner USP, de-represses ecdysone target genes that are high in the early pupal wing such as Broad-Z1 and βFtz-F1 (Ghbeish et al., 2001; Schubiger and Truman, 2000). The EcR/USP heterodimer also cooperates with the SMRTR co-repressor in the wing to prevent precocious expression of ecdysone target genes such as Broad-Z1 (Heck et al., 2012). Consistent with our hypothesis that a repressive EcR/USP complex prevents precocious expression of Broad-Z1 and thereby a precocious G2 arrest, inhibition of SMRTR can also cause a G2 arrest (Pile et al., 2002). Thus, in the context of the early pupal wing, we propose that the significant pulse of ecdysone at the larval-to-puparium transition relieves the inhibition of a repressive receptor complex, leading to Broad-Z1 activation (Fig. 7). Consistent with this model, high levels of Broad-Z1 in the larval wing lead to precocious neural differentiation at the margin (Schubiger et al., 2005) and in our hands precocious inhibition of *stg* expression in the wing pouch (Fig. 5). Interestingly, a switch in Broad isoform expression also occurs during the final cell cycle in the larval eye, such that Broad-Z1 becomes high in cells undergoing their final cell cycle and entering into terminal differentiation (Brennan et al., 2001). However, in this case, Broad-Z1 expression is not associated with a G2 arrest and occurs in an area of high Stg expression, suggesting the downstream Broad-Z1 targets in the eye may be distinct or regulated differently from those in the wing.

The ecdysone receptor has also been shown to down regulate Wingless expression via the transcription factor Crol at the wing margin, to indirectly promote CycB expression (Mitchell et al., 2013). While a loss of EcR at the margin decreased CycB protein levels, the effects of EcR loss on CycB levels in the wing blade outside of the margin area were not obvious (Mitchell et al., 2013). We suggest that in the wing, the role for EcR outside of the margin acts on the cell cycle via a different mechanism through *stg*. Consistent with a distinct mechanism acting in the wing blade, over-expression of Cyclin B in the early prepupal wing could not promote increased G2 progression or bypass the prepupal G2 arrest (Fig. 5). Instead, our results on the prepupal G2 arrest are consistent with previous findings that Stg is the rate-limiting component for G2-M cell cycle progression in the fly wing pouch and blade (Neufeld et al., 1998).

### Gene expression changes during metamorphosis in the wing

In order to identify the gene expression changes in the wing that occur in response to the major peaks of ecdysone during metamorphosis, we performed RNAseq on a time course of pupal wings. We observed major changes in gene expression in this tissue during metamorphosis (Fig. 3, Fig. S3). In addition, we identified known ecdysone targets that are affected differently in the wing during the first larval-to-pupal ecdysone pulse and the second, larger pulse at 24 h APF (Fig. 4). Ecdysone signaling induces different direct targets with distinct kinetics (King-Jones and Thummel, 2005). Furthermore, specific targets, for example Ftz-F1, can modulate the expression of other ecdysone targets to shape the response to the hormone (King-Jones et al., 2005). Thus, we expect that a pulse of ecdysone signaling leads to sustained effects on gene expression and the cell cycle, even after the ecdysone titer returns to its initial state. These factors together with the differences in the magnitude of the ecdysone pulse may contribute to the differences in the response to the early versus later pulses in the wing.

Ecdysone signaling can also affect the cell cycle and cell cycle exit via indirect mechanisms such as altering cellular metabolism. This is used to promote cell cycle exit and terminal differentiation in neuroblasts, where a switch toward oxidative phosphorylation leads to progressive reductive divisions (divisions in the absence of growth) leading to reduced neuroblast cell size and eventually terminal differentiation (Homem et al., 2014). Although reductive divisions do occur in the final cell cycle of the pupa wing (Aigouy et al., 2010, our unpublished data), this type of mechanism does not provide a temporary arrest to synchronize the final cell cycle in neuroblasts as we see in wings. Importantly, we do see a striking reduction in the expression of genes involved in protein synthesis and ribosome biogenesis in the wing during metamorphosis, consistent with the lack of cellular growth (Fig. 3, O'Keefe et al., 2012). Instead the increased surface area of the pupal wing comes from a flattening, elongation and apical expansion of the cells due to interactions with the extracellular matrix creating tension and influencing cell shape changes (Etournay et al., 2015). This is also consistent with our findings that a significant number of genes associated with protein targeting to the membrane are increased as the wing begins elongation in the early pupa (Fig. 3). Further studies will be needed to determine whether the changes in expression of genes involved in ribosome biogenesis and protein targeting to the membrane are controlled by ecdysone signaling, or some other downstream event during early wing metamorphosis.

### Tissue-specific responses to ecdysone can mediate opposite effects on the cell cycle through the same target

Perhaps the most interesting and least understood aspect of steroid hormone signaling is how a diversity of cell-type and tissue-specific responses are generated to an individual hormone. Cell cycle responses to ecdysone signaling are highly cell type specific. For example, abdominal histoblasts, the progenitors of the adult abdominal epidermis, become specified during embryogenesis and remain quiescent in G2 phase during larval stages. During pupal development, the abdominal histoblasts must be triggered to proliferate rapidly by a pulse of ecdysone to quickly replace the dying larval abdominal epidermis. This is in contrast to the behavior of the wing imaginal disc, where epithelial cells undergo asynchronous rapid proliferation during larval stages, but during metamorphosis the cell cycle dynamics become restructured to include a G2 arrest followed by a final cell cycle and entry into a permanently post-mitotic state, in a manner coordinated with tissue morphogenesis and terminal differentiation.

How does the same system-wide pulse of ecdysone at the larval-to-puparium transition lead to such divergent effects on the cell cycle in adult progenitors? Surprisingly it seems to be through divergent effects on tissue specific pathways that act on the same cell cycle targets. In the abdominal histoblasts the larval-to-puparium pulse of ecdysone triggers cell cycle re-entry and proliferation via indirect activation of Stg (Ninov et al., 2009) by modulating the expression of a microRNA miR-965 that targets Stg (Verma and Cohen, 2015) (Fig. 7). This addition of the microRNA essentially allows ecdysone signaling to act oppositely on the same cell cycle regulatory target as Broad-Z1 does in the wing. Thus, tissue-specific programs of gene regulatory networks can create divergent outcomes from the same system-wide hormonal signal, even when they ultimately act on the same target.

## MATERIALS AND METHODS

### Cell culture

Clone-8 (Cl-8) and S2R+ cells were obtained from the Drosophila Genomics Research Center (DGRC, Bloomington, Indiana, USA). A strongly ecdysone-responsive Kc167 subclone was obtain from Dr K. Cadigan (University of Michigan, Ann Arbor, MI, USA). All cells were cultured at 25°C in Schneider's media (Invitrogen) supplemented with penicillin-streptomycin (Gibco) and 10% heat-inactivated ‘Optima’ fetal bovine serum (Atlanta Biologicals). For Cl-8 cells, the media was supplemented with 5 μg ml^−1^ insulin (Sigma) and 2.5% fly extract. Fly extract was prepared and stored as described (https://dgrc.bio.indiana.edu/include/file/additions_to_medium.pdf). Cell counting/viability was performed manually using a hemocytometer and Trypan blue staining. 20-Hydroxyecdysone (Sigma) was dissolved at 1 mg ml^−1^ in dimethyl sulfoxide (DMSO) or water. RNAi experiments were performed in 6-well dishes, with 1-3×10^6^ cells ml^−1^. Cells were seeded for 12-24 h in complete media. Media was removed, then cells were washed with 1 ml 1× PBS and replaced with 0.5 ml serum-free medium containing 10-20 μg ml^−1^ dsRNA overnight. 0.5 ml of complete medium was added and cells were incubated for 3 days prior to flow cytometry or treatment with 20-HE.

Primers used for dsRNA synthesis using T7 Polymerase as described (Rogers and Rogers, 2008):

T7-Wee-fwd, TAATACGACTCACTATAGGGATGACTTTGACAAGGACAC, T7-Wee-rev, TAATACGACTCACTATAGGATCTAGTCGATTGACGCATT; T7-Myt1-fwd, TAATACGACTCACTATAGGAATTGCACGACGACAAACAC, T7-Myt1-rev, TAATACGACTCACTATAGGTGTCCAGATGGATGAGATTC; T7-Myt1-fwd2, TAATACGACTCACTATAGGACAACAATCTGAACCGAAGC, T7-Myt1-rev2, TAATACGACTCACTATAGGTGGAGCCATATACCTCGAAT; T7-EcR-fwd, TAATACGACTCACTATAGGTGCGAAATGGACATGTACAT, T7-EcR-rev, TAATACGACTCACTATAGGTCCCGCGTATATGATCTATT; T7-Br-fwd, TAATACGACTCACTATAGGCTGCAGGATGTCAACTTCAT, T7-Br-rev, TAATACGACTCACTATAGGGTGCTTGATCGTACTGAAGT.

### Flow cytometry

Flow cytometry analysis for DNA content in Figs 1 and 2 and Fig. S1C was performed on live cells in 1× PBS with DyeCycleViolet (Life Technologies) at a 1:2000 dilution for 30 min at room temperature. Cells were run on an Attune Cytometer (Life Technologies) under standard settings using the Violet laser with the 450/40 filter. Flow cytometry analysis for DNA content in Fig. S1A was performed on ethanol-fixed cells, treated with RNaseA (Sigma) and stained with propidium iodide (Sigma) as described (Bettencourt-Dias et al., 2004) and analyzed on a FACSCalibur (BD). For EdU incorporation assays, cells were cultured in complete medium with 10 μM EdU for the indicated timepoints, and either chased with complete media lacking EdU for the indicated timepoints prior to fixation (Fig. 2F), or fixed immediately and stained (Figs 1G and 2A) using the protocol of the Click-IT EdU AlexaFluor-488 Flow Cytometry kit (Life Technologies). For all EdU-treated samples DNA was stained with FX CycleViolet (Life Technologies) for 30 min at room temperature and samples were analyzed on an Attune cytometer. Results from multiple replicates were graphed and analyzed by one-way ANOVA (for comparing groups) or paired *t*-tests (for individual comparisons) using Prism (www.graphpad.com). *P*-values are indicated as follows; *P<0.05, **P<0.01, ***P<0.001, n.s.=*P*>0.05.

### Western blots

Western blots were performed using BioRad TGX precast 4-20% gels, and HRP conjugated secondary antibodies with high sensitivity ECL detection reagents (Thermo) as described (Sun and Buttitta, 2015). We used the following antibodies: anti-Tyr15-P-cdc2 (1:1000, Cell Signaling Technologies, #9111), anti-EcR common (1:1000, DSHB, DDA2.7), anti-Wee (1:700, kindly provided by Dr S. Campbell), anti-Broad Z1 and Broad-Core (1:100, DSHB, #Z1.3C11.OA1 and #25E9.D7), mouse anti-α-tubulin (1:1000, DSHB, AA4.3) and anti-total cdc2 (1:1000, Millipore, #06-923). For Fig. 1, western blot signal was quantified using Image J and presented as the ratio of pCdc2 to total Cdc2.

### Fly stocks

The *w*;*engrailed-Gal4*,*UAS-GFP*; *tub-Gal80TS* and *w*;*apterous-Gal4*,*UAS-GFP*; *tub-Gal80TS* stocks have been described (Buttitta et al., 2007). The *w*;*+*,*act>CD2,stop>Gal4*,*UAS-dsRed* (BL# 30558, Bloomington Drosophila Stock Center) was recombined with the Stg-GFP protein trap (YD0685) kindly provided by Dr Y. Yamashita (University of Michigan, Ann Arbor, MI, USA). EcR RNAi (Schubiger and Truman, 2000), Broad TRiP RNAi lines (BL #27272 and #33641) and UAS-BrZ1 (BL#51379) were crossed to y,w,*hsflp^122^* and are available in the Bloomington Stock Center. The *broad* null allele *npr3* was also used (BL#5964, Bloomington Drosophila Stock Center).

### Immunofluorescence

For EdU labeling, cells were cultured with 10 μM EdU for 45 min in complete medium and fixed and stained using the protocol of the Click-IT EdU AlexaFluor-555 Imaging kit (Life Technologies). DNA was stained for 10 min with 1 μg ml^−1^ Hoechst 33258. For *Drosophila* tissues, pupa were collected and staged at 25°C, using the immobile white-prepupa stage as 0 h APF. Tissues were dissected and fixed in paraformaldehyde, followed by washed in 1× PBS-0.1%Triton-X100 and blocking and staining as described (Buttitta et al., 2007). For Figs 4-6 all samples within each figure were scanned with the same gain and laser intensity settings as the control genotypes. For Fig. 6 GFP signal in the wing pouch (pouch boundary defined by folding at hinge) was quantified (pixels×area) using Image J.

### RNAseq gene expression analysis

Animals were staged at 25°C as described (O'Keefe et al., 2012). Forty wings for each stage were manually dissected and RNA was isolated using Trizol as previously described (McKay and Lieb, 2013). Two independent replicates for each timepoint were performed. Libraries for RNAseq were generated using the stranded mRNA sequencing kit from KAPA Biosystems (catalog # KK8421). Reads were mapped to the genome (dm3) using Tophat2 (Kim et al., 2013) using a transcript annotation file for the alignment. The Htseq-count tool (Anders et al., 2015) was used to count reads mapping to genes, and the EdgeR package in Bioconductor (Robinson et al., 2010) was used to calculate RPKMs. For heatmaps of core cell cycle gene expression across the time series, the percent maximum of TMM normalized read counts was used for hierarchical clustering analysis based on average linkage. For gene ontology analysis, we defined differentially expressed genes as those having a logCPM greater than 2 in at least one sample and changing by at least twofold between pairwise time points. The resulting gene lists were submitted to DAVID (Huang et al., 2009) gene ontology analysis, and subsequently filtered using REViGO (Supek et al., 2011) to remove redundant terms.
